# Recent pattern of Co-infection amongst HIV seropositive individuals in tertiary care hospital, kolkata

**DOI:** 10.1186/1743-422X-8-116

**Published:** 2011-03-14

**Authors:** Kallol Saha, Rushna Firdaus, Poonam Santra, Jyotirmoy Pal, Arnab Roy, Mihir K Bhattacharya, Sekhar Chakrabarti, Provash C Sadhukhan

**Affiliations:** 1 I. C. M. R. Virus Unit, Kolkata, I.D. & B.G. Hospital Campus, GB-4 (East Wing), 1st Floor; 57, Dr. Suresh Chandra Banerjee Road; Beliaghata, Kolkata-700010, India; 2 Institute of Post Graduate & Medical Education and Research, Kolkata; 244, Acharya Jagadish Chandra Bose Road, -700020 Kolkata, India; 3 National Institute of Cholera and Enteric Diseases, Scheme XM, Beliaghata, P-33 C.I.T Road, 700010 Kolkata, India

## Abstract

**Background:**

Opportunistic Infections (OIs) and co-infections are the major cause of deaths amongst HIV infected individuals and this mostly depends upon the risk factors, type of exposure and geographic region. The commonest types of infections reported are tuberculosis, chronic diarrhoea, oral candidiasis, herpes simplex virus-2, cytomegalovirus, hepatitis B virus and hepatitis C virus. Due to the scarcity of OIs data available from this region, we had designed a study to determine the frequency of different OIs amongst HIV seropositive patients.

**Methods:**

Analysis of the different spectrum of OIs/Co-infections were carried out with 204 HIV sero-positive patients (142 males and 62 females) who visited the HIV/AIDS Apex Clinic in a tertiary care hospital from March 2006 to March 2009. The CD4+ count was estimated using FACS Calibur, the routine smear test, serology, nested RT-PCR and DNA sequencing were carried out to determine the different OIs.

**Results:**

In this study, HIV seropositive patients were mostly from middle age group (31-40 yrs) with CD4+ counts in majority of symptomatic AIDS patients below 200 cells/mm^3^. The common co-infections/opportunistic infections were OC (53.43%), CD (47.05%), HSV-2 (36.76%), TB (35.29%), CMV (26.96%), HBV (15.19%) and HCV (7.35%). Dual infections, like HSV-2 & CMV (15.38%), HSV-2 & TB (14.61%), HSV-2 & oral candidiasis (24.61%) and CMV & oral candidiasis (14.61%) were significant in follow-up patients. Triple infections were also common e.g., TB, CD, OC infection occurring frequently in about 14.21% of the study population. Multiple infections like OC, TB, CD amongst the viral co-infected patients with HSV-2, HCV, CMV and HBV are also reported in this study. The genotyping analysis of the HCV co-infected HIV individuals shows that two belonged to HCV genotype 1 and 8 belonged to genotype 3.

**Conclusions:**

A wide spectrum of OIs were observed amongst HIV-infected patients in the HIV/AIDS Apex Clinic. Oral candidiasis, CD, CMV and HSV-2, were the common OIs in those patients. This study aims to provide a clearer picture regarding infections occurring amongst HIV seropositive individuals so that the scientific findings could be translated into sustainable prevention programmes and improved public health policies.

**Trial registration:**

None

## Background

Currently 2.27 million people are infected with human immunodeficiency virus (HIV) with an estimated adult prevalence rate of 0.31% in India alone [1]. OIs lead to frequent morbidity and mortality which shortens the life span of people with HIV infections and requires expensive treatments which becomes a burden for a developing country like India. Timely initiation of prophylaxis of OIs, quick recognition and treatment are the only economically viable options. It has been reported that decrease in CD4+ count is partially responsible for major immunodeficiency's that leads to most of the OIs among HIV infected individuals [2]. The most common OIs/co-infections in HIV infected individuals are tuberculosis, chronic diarrhoea, candidiasis, HSV-2, CMV, HCV and HBV.

HIV is the most important known risk factor that promotes progression to active TB in people with *Mycobacterium tuberculosis *infection. TB can occur at any point in the course of HIV infection. An individual infected with HIV has 10 times more risk of developing TB when compared with a normal person. TB prevalence have also increased 3-5 times in Sub-Saharan Africa in the last decade and HIV seroprevalence has been reported to be about 75% among those patients [3]. It is also very common in India [2,4] due to the similar conditions prevailing here. Oral candidiasis (OC) is a collective term for a group of oral mucosal infection caused by a fungal pathogen candida. It is one of the earliest manifestations of HIV disease and is also a strong predictor of AIDS-related illness or death [5]. Majority of HIV patients usually suffers from chronic diarrhoea (CD) during their period of HIV infection [6]. It is defined as persistence of diarrhoea beyond four weeks. It may be due to either opportunistic or non opportunistic agents, for opportunistic infections leading to chronic diarrhoea a severe, chronic and frequent gastrointestinal disease persists whereas a non-opportunistic agent causes acute but treatable diarrhoeal illness.

Studies related to the occurrence of HSV-2 and CMV among HIV patients from this part of the world are rare, paucity of accurate and relevant data also persists which necessitates the need for better surveillance and public health monitoring. Herpes simplex virus-2 (HSV-2) co-infection is associated with increased genital HIV shedding, which may increase transmissibility of HIV [7]. Genital infection by herpes simplex virus type-2 (HSV-2) is considered one of the major cofactors favouring both sexual transmission and acquisition of the human immunodeficiency virus type-1 (HIV-1) [7]. CMV infection is more prevalent in populations at risk for HIV infection; approximately 75% of injection drug users and >90% of homosexual men who are infected with HIV have detectable IgG antibodies to CMV [8].

Majority of the persons infected with HIV also may occasionally develop severe hepatobiliary problems pertaining to which liver complications like hepatitis B and hepatitis C are on the rise [9,10]. It is reported that among the total cases of HIV infections worldwide, 2-4 million are estimated to have chronic hepatitis B Virus (HBV) co-infection, while 4-5 million are co-infected with hepatitis C virus (HCV) [11]. Co-infection rates of HBV and HCV in HIV patients varies worldwide and largely depends upon the geographic location, risk groups, the type of exposure involved and the socioeconomic condition of that particular region. In Europe and United States of America, HIV-HBV co-infection is around 6-14% [11,12]. It has been shown in recent studies that post highly active anti-retroviral therapy (HAART), HCV related liver disease mortality has increased significantly and is the most common cause of non-AIDS related death amongst HIV patients [13]. There are reports related to HBV and HIV co-infection from different parts of India [9,10] but no such detailed study on HCV and HIV co-infection except some serological observations [14,15].

Timely intervention of OIs not only helps HIV positive persons to live longer but it also helps to prevent transmission of OIs from spreading to others in the community. Since these opportunistic infections and co-infections have a vital role in clinical presentation and is one of the most frequent cause of mortality among HIV patients, the main objective for this study was to elucidate the current frequency and type of the major opportunistic infections/co-infections in HIV seropositive patients in a HIV/AIDS Apex Clinic of a tertiary care hospital of Kolkata. As there were no reports of hospital based HIV and HCV co-infections from this region, we also included this in our study for better management and greater awareness of this dreadful disease and its complications.

## Methods

### Collection of Sample

Over a period of three years i.e., from March 2006 to March 2009, 204 cases of HIV seropositive patients were included in this study from a HIV/AIDS Apex Clinic in IPGME & R, Kolkata, India. Informed consent was obtained before undergoing any kind of test, in case of child patients, consent from their parents were obtained. These patients were from different parts of the Eastern and North Eastern region of India. The HIV testing was performed according to the guidelines of National AIDS Control Organization (NACO), India [1]. Only those HIV seropositive patients willing to participate were included in this study. HIV seropositive's partner were also included after subsequent HIV diagnosis, if they were willing to participate in this study.

### Diagnosis of Opportunistic Infections

The diagnosis of the diseases was done according to the guidelines of Centre for Disease Control and Prevention guidelines (CDC) [2]. The patients who had more than two weeks of cough, blood in sputum and chest pain were only considered for the tuberculosis test. The diagnosis of tuberculosis (TB) was confirmed by microscopic examination of the sputum using acid fast bacilli (Ziehl-Neelsen) staining followed by chest radiogram before coming to a conclusion.

For the conformation of chronic diarrhoea, solely clinical presentations were considered and no microscopic or other types of investigations pertaining to the causal agents were investigated. Oral candidiasis was detected by the clinical presentations and microscopic examination of the yeast forms from the region of oropharynx and this was the sole criterion for the identification of the disease [16].

Viral infections were detected using serological diagnosis like ELISA for HSV-2 (IgM; Equipar, s.r.l, Italy), HBV (ELISA, HBsAg; Biomerieux, Boxtel, Netherlands), CMV (IgM, Equipar, s.r.l, Italy) and HCV (Hepnostica HCV ultra; Biomerieux, Boxtel, Netherlands) according to the manufacturer's instructions.

### Detection of HCV RNA

The HCV seropositive samples were subjected to RNA extraction using QIAamp Viral RNA mini kit (Qiagen, Germany) according to manufacturer's protocol. Detection of HCV viral RNA was done by Nested RT-PCR based on 5'non coding region (NCR) of HCV genome, the primers used in this study were according to Bukh et al [17]. One tube RT-PCR was carried out in a total volume of 20 μl containing 2 μl of Taq 10× buffer II (ABI, USA), 0.8 mM of dNTPs (ABI, USA), 1.5 mM of magnesium chloride, 5 mM of dithiotheritol (DTT) (Sigma-Aldrich, USA), 0.25 μM of the forward and reverse primers, 0.4 U of AMV reverse transcriptase (Promega, USA), 0.5 U of Taq DNA polymerase (ABI, USA) and 2 μl of RNA. The RT-PCR conditions were 42°C for 60 minutes followed by 94°C for 5 minutes followed by 35 cycles of 94°C, 55°C and 72°C for 1 minute each, the final extension step was carried out at 72°C for 5 minutes in an ABI 9700 thermocycler. Followed by nested PCR in a total volume of 25 μl containing 2.5 μl Taq 10× buffer II, 0.8 mM of dNTPs, 2.5 mM of magnesium chloride, 0.25 μM of forward and reverse primers and 0.5 U of Taq DNA polymerase (ABI, USA), and 2 μl of the first round RT-PCR product. PCR conditions were similar as of the RT-PCR, only the RT step i.e, 42°C for 60 was omitted, a band at 256 bp in a 1.5% agarose gel stained in ethidium bromide was observed in gel documentation system (Biorad, USA) in case of HCV positive samples.

### DNA Sequencing and Phylogenetic Analysis

The 256 bp nested PCR amplified products of 5'non coding region of HCV genome were gel purified using Qiaquick PCR purification kit (Qiagen, Germany) according to manufacturer's protocols. The purified products were directly used for DNA sequencing in an automated DNA sequencer (3130XL, ABI, USA) using Big-Dye terminator 3.1 Kit (ABI, USA). The phylogenetic analysis was done using MEGA 4 software and Neighbour-Joining (NJ) method.

The sequences were aligned with the representative number of sequences for each major genotype selected from the Gene Bank database with the help of multiple sequence alignment software (MEGA 4). 181 bp (nt-240 to -60) of the 256 bp product was used for phylogenetic analysis. The evolutionary history was inferred using the Neighbour-Joining method [18]. The optimal tree with the sum of branch length = 0.30199712 is shown. The percentage of replicate trees in which the associated taxa clustered together in the bootstrap test (2000 replicates) is shown next to the branches [19]. The tree is drawn to scale, with branch lengths in the same units as those of the evolutionary distances used to infer the phylogenetic tree. The evolutionary distances were computed using the Maximum Composite Likelihood method [20] and are in the units of the number of base substitutions per site. All positions containing gaps and missing data were eliminated from the dataset (complete deletion option). There were a total of 133 positions in the final dataset. Phylogenetic analyses were conducted in MEGA4 [21]. Pairwise comparisons for evolutionary distance for percent nucleotide homology were made by Jukes-cantor algorithms.

### Determination of CD4

The CD4+ counts of the HIV seropositive persons were estimated using FACS CALIBUR flow cytometer (Becton Dickinson, California, USA). Standard dual colour immunophenotyping method was performed using whole blood.

### Statistical Analysis

The statistical analysis was performed using student's t test. The Null Hypothesis was also tested and a P value < 0.05 was considered to be statistically significant. Mean, median and mode were also estimated.

## Results

### Demographic profile

**Figure 1:**
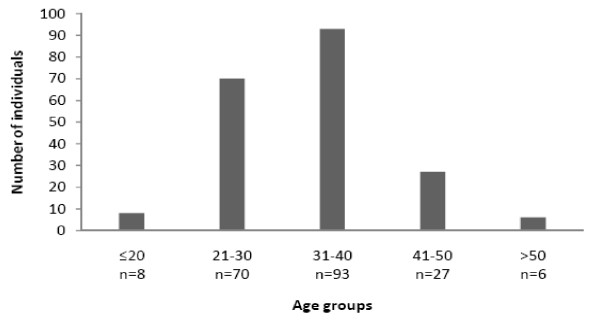
**Distribution of the patients of opportunistic infection with age group**.

**Figure 2:**
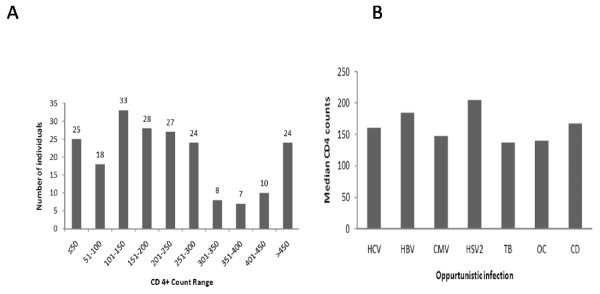
**Pattern of CD4+ counts and the respective median CD4+ value of different OIs among the HIV seropositive individuals.** A: Distribution of CD4 count in the study population. The varying levels of CD4 counts in the sample study is shown in the alongside figure the y axis shows the no. of persons and the x axis represents the class interval CD4 value with an interval of 50 CD4 count. B: Median CD4 distribution with the various opportunistic infections. HCV: Hepatitis C Virus; HBV: Hepatitis B Virus; CMV: Cytomegalovirus; HSV-2: Herpes Simplex Virus 2; TB: Tuberculosis; OC: Oral Candidiasis; CD: Chronic Diarrhoea.

In this study, HIV positive patients were mostly from middle age group (Figure 1),CD4+ counts in majority of symptomatic AIDS patients were below 200 cells/mm^3^(Figure 2A). Most of the patients were from age group 20-40 (79.9%) years. Male gender were predominant with 142 (69.60%) representatives and 62 (30.4%) females, the age group ranges from 9 yrs to 66 yrs with the age group 31-40 yrs (43.62%) having the largest number of affected individuals (Figure 1). Migrant labourers were the commonest occupational group followed by truck drivers and constituted a considerable proportion of the study. These migrant workers were largely employed as goldsmiths in Mumbai and Pune (Western region of India). The most identifiable risk factors were extra-marital heterosexual contact and had history of exposure to commercial sex workers. Women were generally the victims of their husbands. None of the patient was reported homosexual practices. Generally most of the cases were referred by medical practioners or approached on their own, largely the patients came to the clinic with common complaints of fever, weight loss and diarrhoea, and subsequent investigation revealed them to be HIV seropositive. Due to the clinic being a tertiary care centre hence the true proportional representation of the population could not be accurately ascertained. Only two individuals had HIV infection from blood transfusion, whereas rest all had been infected by heterosexual means.

### Epidemiological Findings

**Figure 3:**
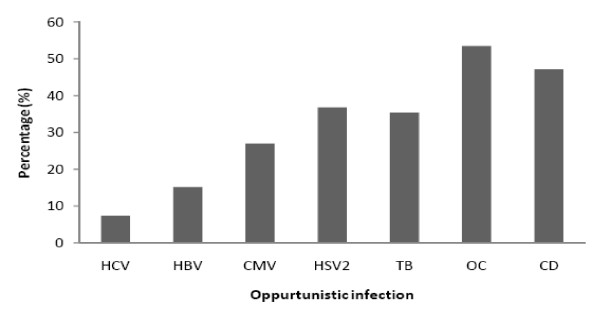
**Distribution of various opportunistic infections among HIV infected patients**. HCV: Hepatitis C Virus; HBV: Hepatitis B Virus; CMV: Cytomegalovirus; HSV-2: Herpes Simplex virus 2; TB: Tuberculosis; OC: Oral Candidiasis; CD: Chronic Diarrhoea.

In this study, all patients were infected with HIV-1, out of 204 patients examined 109(53.43%, median CD4+ counts 160 cells/mm^3^) were co-infected with oral candidiasis,96 (47.06%, median CD4+ counts 167 cells/mm^3^) had chronic diarrhoea, 75(36.76%, median CD4+ counts 204 cells/mm^3^) were HSV-2 IgM positive, 72(35.29%, median CD4+ counts 137 cells/mm^3^) had tuberculosis, 55 (26.96%, median CD4+ counts 147 cells/mm^3^) were CMV IgM positive, 31 (15.19%, median CD4+ counts 147 cells/mm^3^) were HBsAg positive and 15 (7.35%, median CD4+ counts160 cells/mm^3^) were positive to HCV antibody (Figure 2B and 3). 74.5% of the co-infected cases were accompanied with fever. Seventeen (8.33%, median CD4+count 481 cells/mm^3^) individuals had no history of co-infection (3 male and 14 female), among the three affected males, one had been infected from his wife. All the asymptomatic HIV positive female patients were infected from their husbands.

### Multiple Co- Infections

**Figure 4:**
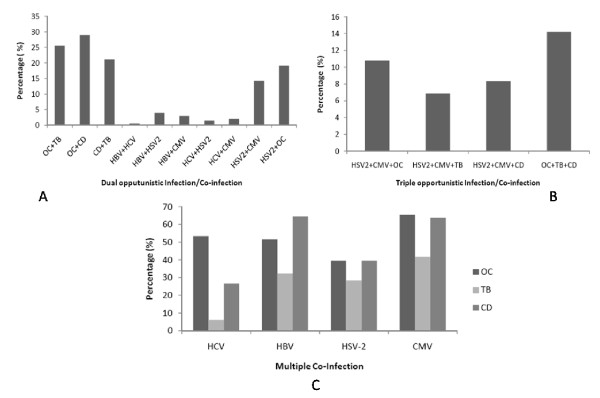
**Multiple co-infections among HIV seropositive individuals.** A: Frequency of Dual opportunistic infection and Co-infections in HIV seropositive individuals. OC: Oral Candidiasis; TB: Tuberculosis; CD: Chronic Diarrhoea; HBV: Hepatitis B Virus; HCV: Hepatitis C Virus; HSV-2: Herpes Simplex virus 2; CMV: Cytomegalovirus. B: Frequency of triple opportunistic Infection and Co-infections in HIV seropositive individuals. HSV-2: Herpes Simplex virus 2; CMV: Cytomegalovirus; OC: Oral Candidiasis; TB: Tuberculosis; CD: Chronic Diarrhoea. C: Opportunistic infections in different viral co-infected HIV seropositive patients. The representation of the multiple co-infection among the HIV positive study population with viral co-infection. HCV: Hepatitis C virus; HBV: Hepatitis B Virus; CMV: Cytomegalovirus; HSV-2: Herpes Simplex virus 2; OC: Oral Candidiasis; TB: Tuberculosis; CD: Chronic Diarrhoea.

The important observations of this study were dual, triple opportunistic infections and co-infections among the HIV seropositive individuals. The most frequent dual infections were CD and OC 28.92%, OC and TB 25.49%, CD with TB 21.08%, HSV-2 and OC 19.11%, HSV-2 and CMV 14.21%, HBV and HSV-2 3.92%, HBV and CMV 2.94%, HCV and CMV 1.96%, HCV and HSV-2 1.47%, and the least prevalent one was HCV and HBV 0.49% infection which showed only one case (Figure 4A). The commonest triple infection were OC, TB, CD at 14.21%, HSV-2, CMV, OC at 10.78%, HSV-2, CMV, CD at 8.34% and HSV-2, CMV, TB at 6.86% respectively (Figure 4B). Apart from this, the trend of infection with TB, OC and CD among HCV, HBV, HSV-2 and CMV co-infected individuals is shown in Figure 4C.

The frequency of occurrence of multiple viral infection were less when compared to that of the other bacterial and yeast infection i.e., among the HBV infected population only 29.03% had HSV-2 co-infection, 16.12% had CMV co-infection and 3.2% had HCV co-infection; among HCV infected population 6.67% had HBV co-infection, 20% had HSV-2 co-infection and 26% had CMV co-infection respectively (Figure not shown).

There was only one toxoplasmic encephalitis case diagnosed at a later stage of an AIDS patient and subsequently the patient died. Three patients showed Varicella Zoster Virus (VZV) clinical presentation but was not confirmed with serology. There was not a single case of *Pneumocystis sp*. related pneumonia in the study population.

### HCV Genotyping

**Figure 5:**
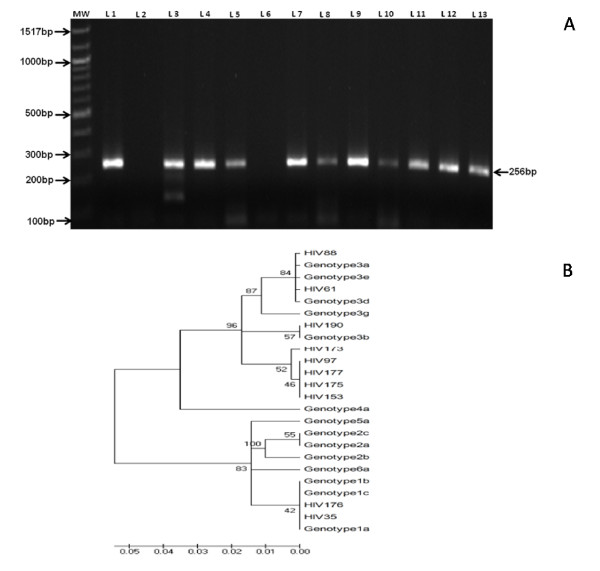
**Electrophoretic pattern of nested RT –PCR and phylogenetic tree of HCV RNA positive individuals.** A: Agarose gel picture of nested RT- PCR amplified product of 5'NCR of HCV genome. Lane 1: MW (100 bp DNA ladder); lane 2: positive control; lane 3: negative control; lane 3, 4, 5, 7, 8, 9, 10, 11, 12, 13 depicts the positive sample numbered HIV35, HIV61, HIV88, HIV97, HIV153, HIV173, HIV 175, HIV 176, HIV177 and HIV 190 respectively. B: Phylogenetic analysis of hepatitis C virus 5' non coding region. Phylogenetic analysis of 5' non coding region (nt -240 to -60; 181 bp) sequences of 10 HCV samples of HIV seropositive individuals. The sequences for major subtype were selected from the GeneBank database for analysis. The accession Numbers of the reference sequences (with subtypes are as follows: AF011753 (1A), AJ132996 (1B), AY051292 (1C), af238485 (2a), af238486 (2b), L38330 (2c), AF046866 (3a), D49374 (3b), D16612 (3c), D16620 (3d), D16618 (3e), X91421 (3g), Y11604 (4a), Y13184 (5a), Y12083 (6a) and the samples are numbered as HIV following the reference number.

Among the 15 HCV seropositive HIV individuals, 10 were HCV RNA positive (4.90%, median CD4 count 74 cells/mm^3^) (Figure 5A). On the basis of DNA sequencing and phylogenetic analysis, two out of ten samples were classified as HCV genotype 1 (20%) and eight belonged to HCV genotype 3 (80%) (Figure 5B). There was no correlation with CD4 count and HCV genotype.

## Discussion

In our study, majority of HIV seropositive patients were (31-40 yrs) in their active sexual stage and most of them were detached from their family for long time. Most of the males were migrant workers or drivers, most female HIV patients were their life partner. Transfusion related HIV acquisition was only ~1% in our study.

Oral candidiasis associated with HIV infection occurs frequently and could be considered as an initial manifestation of the disease. Oropharyngeal candidasis (OPC) has been reported to occur in 50 to 95% of all HIV-positive persons at some point during their progression to full-blown AIDS [22]. Oral candidiasis may be considered as a clinical surrogate for severe CD4+ depletion. However, the absence of OC does not necessarily exclude severe immunosuppression. Our data indicates that 53.43% of the individuals were affected with OC. A noteworthy observation in the present study is that the patients who presented HIV associated oral candidiasis without any type of Anti Retroviral Therapy (ART) had a median CD4+ count lower than 200 cells/mm^3^, an associated inverse relationship between CD4+ count and the prevalence of OC could be inferred. The debilitating impact of HIV infection on mucosal Langerhan's cells and CD4+ population were most probably central to the increased pathogenesis of mucosal candidiasis in HIV patients [23]. These findings could definitely help our clinicians to use OC as a helpful tool for the diagnosis and detection of progression of HIV in a resource poor country like India. Thus the present study depicts OC as the predominant OIs amongst HIV affected individuals in this part of India.

Major studies from India [24-27] reports chronic diarrhoea, gastrointestinal infection as the most common OIs associated with HIV patients but our study revealed it to be the second major complication following oral candidiasis whose median CD4+ count was estimated to be around 167 cells/mm^3^. Organisms causing chronic diarrhoea are usually self-limiting but as the end stage of AIDS approaches they are known to be life threatening too. Patients with multiple intestinal infections accompanied with profound and persisting diarrhoea have low CD4+ T-cell counts. The number of undiagnosed patients with CD in a developing and resource poor country like ours is on the increase and needs to be properly addressed for better management of HIV related mortality. Immunodeficiency increases the risk of having opportunistic parasites and diarrhoea; therefore it is imperative that health care providers specifically evaluate their HIV infected patients for diarrhoea to increase their quality of life. Since the study had certain limitations with regard to the detailed examination of the stool samples hence the exact etiological agents were not verified as common etiologic agents have already been reported from various parts from India including from this region [28].

TB is one of the most common type of OIs associated with HIV as reported in a study conducted in north-India [2] and this may be associated with endemic factors such as malnutrition, poor hygiene, poverty, migrant population and unemployment which are widely prevalent in this area. In our study, 35.29% of the patients were diagnosed with tuberculosis which was not surprising as TB (median CD4+, 137 cells/mm^3^) is one of the disease which is increasing in India and our data is in accordance with the other published data from this part of India [21,22]. The whole process of diagnosis of TB was well planned so that the number of false negatives could be reduced as the detection of TB among HIV individuals is very difficult. The patients having more than 2 weeks of cough, chest pain and blood along with sputum underwent the sputum microscopy test, the negative samples underwent a generalized antibiotic treatment for 2 weeks so that the other causative agents responsible for the outcome of such type of disease other than TB could be cured, after the antibiotic treatment they again underwent the sputum microscopy test and if the results came out to be negative, chest radiograph was used for the final conclusive result. This process of detection helped us to minimize errors to a large extent.

Herpes simplex virus-2 (36.76%) infection was the most common viral co-infection and this was largely due to the common risk factors. The characteristic features of HSV-2 infection are symptomatic reactivation and asymptomatic viral shedding. Infection with HSV-2 is a lifelong burden as the virus becomes permanently latent in the nerve node ganglion corresponding to the site of inoculation. HSV-2 induces cell mediated immune response hence a higher titre of median CD4+ counts of 204 cells/mm^3 ^was observed in our findings. The arguments pertaining to the co-infection of HSV-2 and HIV includes sexual accusition, transmission which includes disruption of mucosal integrity and in vivo association between the genital RNA load of HIV and DNA load of HSV-2 amongst both asymptomatic and symptomatic shedders [29]. Patients with AIDS, progressive loss of immune function, and in particular loss of cell-mediated immunity, permits CMV reactivation and replication to begin; asymptomatic excretion of CMV in urine could be detected in approximately 50% of HIV-infected individuals with a CD4+ lymphocyte count <100 cells/mm^3 ^[30]. In our study, 26.96% of the patients reported having cytomegalovirus co-infection with median CD4+ counts 147 cells/mm^3^. Cytomegalovirus is reported to be largely dependent upon the type of transmission of HIV and seen largely to occur among homosexuals and since our study reports no cases of homosexuality hence this low frequency of occurrence could be validated [31]. Not a single patient was found with Kaposi's sarcoma which is very frequent with CMV infection.

The available literature suggests a very variable rate for the co-infections of HCV and HBV among the HIV subjects [9,32,33]. It has been stated that HIV has a direct effect on the life cycle of HBV and also a very impounding effect on the host's ability to clear the infection and if this was to be true then we could expect more number of cases with severe chronic liver damage and hepatocellular carcinoma (HCC). Thus the public health aspect must be looked into as this could become one of the realities of the future as the very cause and effect of immunodeficiency on HBV has not been studied yet [10]. Our data shows 15.19% of these populations were infected with HBV (HBsAg) and this corroborates with other published data from India [14]. Since HBsAg signifies the active form of the virus hence HBV DNA detection was not done on HBsAg positive individuals.

The frequency of the HCV infections among our study group was 7.35% (serology), which is fairly constant with other findings from this part of the world [10] whereas, active HCV infection i.e., HCV RNA positive was 4.90% (median CD4+ count 74 cells/mm^3^). There are variable HIV-HCV co-infection reports from other parts of India which ranges from 4.8% - 21.4% in Southern part [34,35], 30% in Western India [36] and the highest is reported from North-East which is about 92% (serology) [15].

Among the ten HCV RNA positive samples, eight were belonged to HCV genotype 3 and two belonged to genotype 1 based on DNA sequencing and phylogenetic analysis of the 5'non-coding region of the HCV genome. We could not find correlation with CD4+ count and HCV genotype. History of HCV infection was not transfusion related, probably it may be from other routes of transmission. HCV genotyping study was considered because of its great epidemiological significance and has not been adequately ascertained in Indian context among HIV population. HCV genotype 3 is the most prevalent form in Northern and Eastern part of India [37,38]. In Western region significant difference in HCV genotype 3 and genotype 1 is not observed [36,38,39] but in Southern India, HCV genotype 1 is more predominant than Genotype 3 [34,36,38]. Compared to HCV monoinfection, HCV associated with HIV shows more rapid progression to cirrhosis, decompensated liver diseases, hepatocellular carcinoma and sometimes death. Cirrhosis may suppress the immunity and may affect the CD4+ count [13]. In our study, median CD4+ count of HCV (RNA positive) co-infected HIV patients was lowest and this corroborates with other study [13]. In cases of advanced HIV, HCV ELISA may result into false negatives and hence the HCV RNA PCR becomes one of the sole criterions for confirmation or exclusion of active disease. Quantitative HCV viral load does not determine the degree of liver damage and hence the genotyping was performed [39]. Since there is no detailed documented report of HCV infection among HIV patients in tertiary care hospital from this region, so a study was needed for better HCV management among HIV infected individuals and this is first such report. Similar to HBV the immunosuppressive action of HIV is also not known on HCV and the further implications which could be associated with it needs to be ascertained.

## Conclusions

Opportunistic infections continues to be one of the most universal complication of HIV infected patients and the public health efforts to curb HIV infection should be in the limelight, as the very importance of precluding and treating HIV related co-infections in India is likely on the increase. The overall frequency of opportunistic infections is very high (53.43%) when compared to data from other parts of India (24, 25, 31, 35). This study depicts a broad range of co-infections associated with HIV infected persons and could be used very aptly in any such resource limited country like India where these data could help improve the public health status of the population. A complete comprehensive population based data comprising four different regions or a larger region of India must be initiated to properly verify the actual cause of this very variable type of occurrence of OIs and make database of infected individuals with HIV so that a clear picture of the OIs is obtained.

There were some limitations of the study as we looked into the clinical presentation of the disease in a hospital and accordingly the incidence rate; prevalence rate could not be deducted from these findings as they were solely from a hospital and not from a general population. We also did not look into the type of multiple drug resistant strains present among the tuberculosis patients within the spectrum of study subjects. The different types of etiological agents causing diarrhoea were also not looked into. Development of resistance remains under studied and hence more works needs to be done in this area. There is an urgent need to interpret the scientific findings into sustainable prevention programmes and improve public health policy. Interventions aimed at preventing and treating HIV associated OIs forms an integral component for maintaining and improving the living standards of those infected. More research on the effectiveness of less expensive interventions needs to be done in resource poor settings like India. Our findings aim to help our clinicians to use OIs for early diagnosis and detection of the progression of HIV infection

## Competing interests

The authors declare that they have no competing interests.

## Authors' contributions

The authors KS, RF, PS and PCS made the study design, implementation, scientific studies and all the experimental verification. The authors JP and AR were involved in first line interaction with the patients and the author MKB and SC helped on laying out the field work. KS, RF and PS wrote the manuscript and PCS made corrections and provided valuable inputs regarding the study. All the authors have read and approved the final manuscript.
